# The Gut Microbiome in Multiple Sclerosis

**DOI:** 10.1212/NXI.0000000000200638

**Published:** 2026-08-26

**Authors:** Alicia Sánchez-Sanz, Sergio E. Baranzini

**Affiliations:** From the Department of Neurology, Weill Institute for Neurosciences, University of California, San Francisco.

## Abstract

Multiple sclerosis (MS) is a chronic inflammatory demyelinating disease of the CNS whose risk and course are shaped by both genetic and environmental factors, among which the intestinal microbiota has emerged as a key, potentially modifiable contributor. People with MS frequently display altered gut microbiota, characterized by depletion of fiber-fermenting, short-chain fatty acid (SCFA)–producing commensals, and expansion of taxa associated with mucus degradation and proinflammatory metabolism. These compositional shifts are paralleled by broad changes in blood and CSF metabolites, including reduced SCFAs, tryptophan-derived aryl hydrocarbon receptor ligands, and secondary bile acids. In addition, several reports highlight the accumulation of aromatic amino acid–derived phenolic and indolic compounds and specific lipid mediators linked to neuroinflammation and neurodegeneration. In this up-to-date review, we synthesize evidence from experimental models and human studies showing how microbial metabolites can influence MS pathogenesis through converging mechanisms: modulation of gut and blood–brain barrier integrity; shaping of T-cell and B-cell responses; direct effects on microglia, astrocytes, and oligodendrocyte lineage cells after crossing into the CNS; and modulation of neural circuits, particularly those involving the vagus nerve. Finally, we highlight current gaps, including the need for longitudinal, harmonized multiomic cohorts, and mechanistic studies integrating microbiome, metabolome, and host readouts across gut, blood, and CNS. Overall, available data support a model in which coordinated disruption of the gut microbiota–metabolite axis, rather than any single pathogen, contributes to MS, opening avenues for microbiota-based and metabolite-based biomarkers and therapies aimed at restoring immune and neuroglial homeostasis.

## Introduction

### The Gut Microbiota as a Modifiable Environmental Contributor to MS

Multiple sclerosis (MS) is a chronic, immune-mediated disease of the CNS characterized by inflammatory demyelination and neurodegeneration. Although susceptibility is strongly influenced by host genetics, concordance rates in monozygotic twins and the increasing incidence of MS in many regions suggest a significant contribution from environmental factors. Commonly implicated exposures include Epstein–Barr virus, vitamin D, smoking, and obesity, yet these do not fully explain interindividual variability in disease risk, course, and progression.^1^ This has fueled interest in the gut microbiota as an additional, potentially modifiable contributor to CNS autoimmunity. The gut microbiota interacts with the host immune, metabolic, and nervous systems, shaping a “gut–brain axis” that can influence distant organs, including the CNS. Importantly, this factor is modifiable—evidence suggests that altering the microbiota (through diet, probiotics, or fecal transplantation) can potentially alleviate MS symptoms or influence disease course.^2^

In this review, we summarize current evidence linking the gut microbiota to MS, with a particular focus on microbial metabolites as mechanistic mediators. We first outline the main routes of gut–CNS communication, then review recurrent microbiome signatures in MS, and finally discuss the major metabolite classes implicated in disease pathogenesis. An overview of these interconnected pathways is summarized in Figure.

**Figure:**
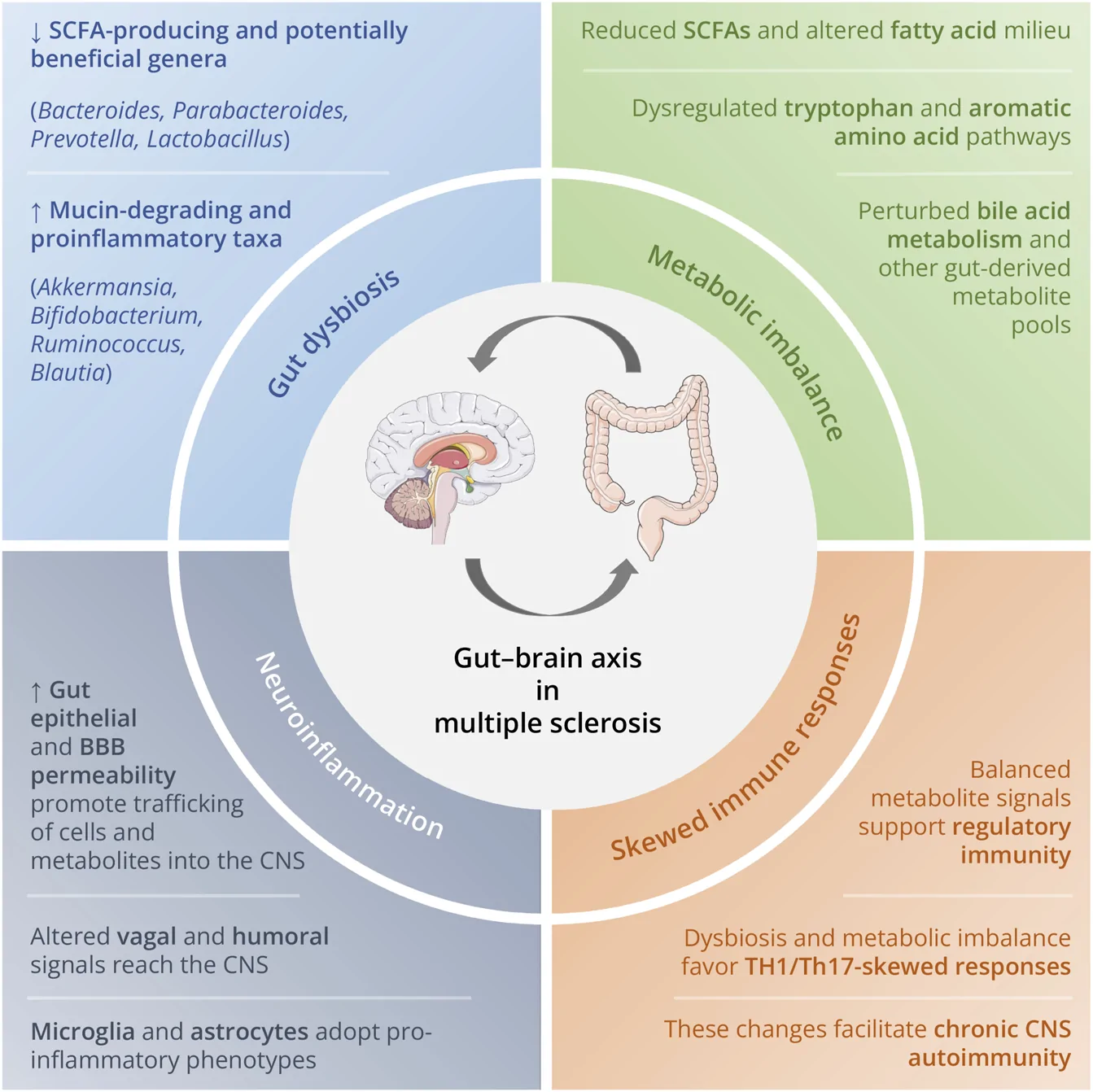
Overview of the Gut–Brain Axis in Multiple Sclerosis MS-associated dysbiosis involves a taxonomic shift, with a relative loss of several commensals commonly linked to anti-inflammatory metabolism (including SCFA-associated taxa) and an expansion of mucus-adapted and/or proinflammatory bacteria. These community changes are accompanied by altered microbial metabolite pools (e.g., reduced SCFAs and perturbed bile acid and tryptophan-derived pathways) and immune skewing (Th1/Th17-biased responses vs regulatory programs). Together, they may impair intestinal and BBB integrity and influence neural/humoral signaling, facilitating immune trafficking and proinflammatory activation of CNS-resident cells that reinforces neuroinflammation. Image(s) provided by Servier Medical Art (smart.servier.com), licensed under CC BY 4.0 (creativecommons.org/licenses/by/4.0/). BBB = blood–brain barrier; SCFA = short-chain fatty acid.

## Gut–CNS Connections

### Vagal and Circulating Pathways

The vagus nerve is a major neural pathway of the gut–brain axis, providing bidirectional communication between the gut and the CNS, while circulating microbial metabolites and hormones serve as complementary humoral messengers. Through these channels, gut-derived signals can reach the CNS, influencing neurologic function and inflammation.

The vagus nerve can modulate inflammatory responses through cholinergic signaling. In the experimental autoimmune encephalomyelitis (EAE) model, vagotomy has been reported to ameliorate disease severity, an effect associated with reduced acetylcholine release and altered CD4^+^ T-cell proliferation and differentiation.^3^ Similarly, loss of choline acetyltransferase in Th17 cells attenuates disease progression, supporting a pathogenic role for Th17 cell-intrinsic cholinergic signaling in EAE.^4^ At the same time, electrical vagus nerve stimulation can also ameliorate EAE, decreasing immune cell infiltration, myelin damage, blood–brain barrier (BBB) disruption, and proinflammatory microglial activation, suggesting that the effects of vagal manipulation may depend on the type of intervention and downstream pathway engaged.^5^ Moreover, studies in nonautoimmune demyelinating models also implicate vagal pathways in CNS pathology. In a lysolecithin-induced focal demyelination model, vagus nerve stimulation reduced innate neuroinflammation and enhanced remyelination, whereas in cuprizone-treated mice, subdiaphragmatic vagotomy ameliorated demyelination and microglial activation and partially normalized gut microbiota composition and metabolite profiles.^6,7^ In people with MS (pwMS), the relationship between vagal activity, gut microbiota, and disease pathophysiology remains unexplored.

In parallel, microbial metabolites may provide a humoral pathway for gut-derived modulation of the CNS. Short-chain fatty acids (SCFAs) can enter the bloodstream and influence microglial maturation and inflammatory tone, while tryptophan-derived metabolites can modulate microglial and astrocyte activity.^8,9^ Moreover, dysbiosis-associated activation of the host kynurenine pathway may promote accumulation of neurotoxic metabolites such as quinolinic acid, which has been linked to neuroinflammation and demyelination in EAE.^10^ Altogether, these experiments suggest that vagal and humoral routes enable the gut microbiota to affect CNS function in the absence of direct microbial translocation.

### Immune Cell–Microbiome Interactions

Growing evidence indicates that gut microbes shape systemic immunity by skewing T cells and other immune cells, which can then traffic to the CNS.^11^ In MS, gut microbiota alterations have been linked to a proinflammatory immune profile, including enhanced Th1/Th17 activity, whereas commensal bacteria can also modulate immune responses through immunomodulatory metabolites.^12^ Molecular mimicry between microbial peptides and myelin antigens may further contribute to autoimmune activation.^13^

EAE models provide compelling evidence that the gut microbiota is required for CNS autoimmunity. Germ-free (GF) mice exhibit attenuated disease with reduced Th1/Th17 responses, whereas colonization with commensal microbiota through fecal microbiota transplant (FMT) restores their susceptibility to EAE and increases intestinal and spinal cord Th1 and Th17 cells.^11,12^ Importantly, colonization with microbiota from pwMS exacerbates EAE and reduces regulatory T-cell (Treg) frequency.^14^ Similarly, FMT from monozygotic twin pairs discordant for MS into transgenic mice with spontaneous brain autoimmunity showed that transplantation of MS-derived microbiota increased disease incidence and was associated with reduced IL-10–mediated regulation.^15^ These findings are consistent with evidence that encephalitogenic T-cell programs are primed in the gut prior to CNS infiltration: MOG-reactive CD4^+^ T cells and Th17 cells appear in the small intestine before CNS infiltration, and gut-associated lymphoid tissues such as Peyer patches can promote the emergence of pathogenic γδT17 cells that migrate to the CNS.^16,17^

Microbiota-targeted interventions further support this link. In EAE and cuprizone models, antibiotic-mediated depletion ameliorates disease severity and reshapes microbial communities toward taxa that promote Tregs, while probiotic approaches (e.g., Lactobacillus spp. and Prevotella histicola) reduce disease through immunoregulatory mechanisms, including increased Tregs, tolerogenic dendritic cells, and suppression of Th1/Th17 responses.^e1-e5^

In humans, pwMS show increased intestinal Th17 cells associated with disease activity and microbiota shifts, including enrichment of Th17-promoting taxa and depletion of anti-inflammatory commensals.^18^ PwMS also harbor microbiota linked to impaired regulatory immune function, including reduced IL-10–dependent T-cell regulation, while probiotic supplementation can promote a regulatory immune phenotype with increased Tregs and reduced Th1 responses.^19,20^ Although human data in the CNS remain limited, translational evidence suggests that microbiota-shaped T-cell programs extend to this compartment. Gut microbiota–dependent CCR9^+^CD4^+^ memory T cells are altered in secondary progressive MS (SPMS) and detectable in the CSF during relapse, and in EAE these cells infiltrate the CNS and are reduced by antibiotic treatment, supporting a link between gut-primed T-cell programs and CNS immune activity.^e6^ Microbiota–immune interactions also extend to the B-cell compartment. In EAE, gut-derived IgA^+^ B cells can migrate to the CNS and exert IL-10–dependent regulatory effects, while in pwMS, microbiota-specific IgA^+^ B cells and reduced IgA coating of gut bacteria have been associated with disease activity, relapse-related CSF IgA changes, and impaired immune tolerance.^e7-e10^ Although direct evidence linking microbiota perturbations to innate immune cell changes within the CNS compartment remains limited, EAE studies indicate that microbiota-modifying interventions can alter CNS inflammatory infiltrates, including monocyte-macrophage and dendritic cell populations.^21^ In addition, experimental studies have shown that microbiota-derived signals can modulate microglial activation, further supporting a link between gut-derived cues and the CNS immune microenvironment.^9^

Collectively, these findings highlight bidirectional crosstalk between the gut microbiota and adaptive immunity, linking intestinal immune modulation to peripheral and CNS inflammation in MS.

### Intestinal Epithelial and Blood–Brain Barriers

The intestinal epithelium and the BBB are key interfaces that mediate gut–CNS crosstalk. Increased intestinal permeability (“leaky gut”) has been reported in MS and may facilitate abnormal immune activation.^22^ Gut bacteria can also influence BBB integrity, highlighting how barrier dysfunction could connect peripheral microbial signals to central inflammation.^23^

During EAE, increased intestinal permeability and morphological alterations of the gut barrier are detectable even before symptom onset.^24^ Modulating the microbiota with oral vancomycin preserves intestinal barrier integrity by reducing trypsin activity and gut permeability, which has been associated with attenuated EAE severity.^25^ Regarding the BBB, GF mice display increased permeability and disorganized tight junction proteins, whereas administration of SCFA-producing bacteria can reverse these effects.^23^

In pwMS, evidence for increased epithelial permeability is suggestive but inconsistent across studies. Circulating zonulin—a proposed regulator of paracellular permeability—was unchanged in 1 cohort and did not vary with disease activity.^26^ By contrast, another study reported higher circulating zonulin levels in pwMS than in healthy controls (HCs).^27^ In an independent cohort, zonulin and intestinal fatty acid–binding protein (a marker of enterocyte injury/transcellular leakage) were similar between pwMS and HCs, yet zonulin correlated with BBB disruption and was higher in pwMS with gadolinium-enhancing lesions.^28^ Several reports have also described increased circulating tight-junction–related proteins in pwMS, but these blood measurements remain indirect proxies of barrier status.^29,30^

Functional readouts are limited and not fully concordant: Small studies have reported increased permeability using the lactulose–mannitol absorption test, but replication in larger, well-controlled cohorts is still needed.^22,31^ Beyond paracellular/transcellular markers, the mucus barrier may also be compromised in MS: Intestinal mucin has been reported to be reduced in relapsing-remitting MS (RRMS) and progressive MS and to correlate with increased abundance of mucus-degrading taxa (e.g., Akkermansia, Bacteroides, and Ruminococcus).^29^ Finally, 1 report showed that pwMS have a higher prevalence of Clostridium perfringens strains producing epsilon toxin, which can kill oligodendrocytes in culture and disrupt the BBB, facilitating immune-cell entry.^32^

Together, these findings suggest that the gut epithelium and BBB act as coordinated gatekeepers of immune–CNS homeostasis, and that microbial imbalance may disrupt this dual barrier system, enabling peripheral inflammatory signals to amplify neuroinflammation in MS. Experimental models provide mechanistic support for this concept, whereas human data remain heterogeneous and largely associative.

## Bacterial Taxa Associated With MS

### Common Gut Microbiome Alterations in MS

Certain microbial changes have been observed across MS cohorts, and current evidence supports a consistent ecological shift rather than a single “MS bug.” PwMS exhibit a characteristic reshaping of the intestinal microbiota, characterized by loss of fiber-fermenting, anti-inflammatory microorganisms (e.g., SCFA producers) and enrichment of taxa linked to proinflammatory metabolism and mucus degradation, which may promote mucosal inflammation. Key MS-associated taxa are summarized in Table 1, while a detailed list of genera and species, including directionality and study support, is provided in eTable 1.

**Table 1:** Key Gut Bacterial Taxa Associated With Multiple Sclerosis

Taxon	Direction in MS	Proposed function	Evidence type	Consistency of findings
Akkermansia muciniphila	↑	Mucin degradation; barrier modulation; context-dependent metabolic and immune effects	Human + experimental	Recurrent
Bacteroides spp. (including B. fragilis)	↑/↓	Polysaccharide and bile acid metabolism; strain-dependent immunomodulation	Human + experimental	Inconsistent
Blautia spp.	↑/↓	SCFA production; immunomodulation (strain-dependent)	Human + experimental	Inconsistent
Eggerthella lenta	↑	Immune modulation; associated with inflammation	Human	Limited evidence
Eubacterium spp.	↓	SCFA (butyrate) production; immunoregulatory effects	Human	Consistent
Faecalibacterium prausnitzii	↓	Butyrate producer; anti-inflammatory; promotes Tregs	Human + experimental	Consistent
Prevotella spp. (including P. Copri)	↓	Fiber degradation; anti-inflammatory effects; Treg induction	Human + experimental	Mostly consistent
Roseburia spp.	↓	SCFA (butyrate) production; gut barrier support	Human	Consistent
Ruminococcus spp. (e.g., R. gnavus, R. torques)	↑	Mucin degradation; associated with barrier disruption and proinflammatory activity	Human	Recurrent
Streptococcus spp.	↑	Th17-promoting activity; associated with proinflammatory immune responses	Human	Recurrent
Tyzzerella nexilis	↑	Associated with progression; enriched in mobile genetic elements; shown to exacerbate EAE in experimental models	Human + experimental	Emerging

Abbreviations: EAE = experimental autoimmune encephalomyelitis; MS = multiple sclerosis; SCFA = short-chain fatty acid.

Consistency of findings reflects the reproducibility of reported associations across studies, accounting for variability related to strain-level differences and cohort-specific factors.

In predominantly RRMS and mixed cohorts enriched for relapsing disease, a recurrent feature is depletion of primary degraders of plant polysaccharides and secondary SCFA-producing commensals, including taxa within Prevotella, Bacteroides, Parabacteroides, Faecalibacterium, Roseburia, Anaerostipes, and Eubacterium.^14,33,34,e11-e26^ These shifts indicate disruption of the fiber–SCFA axis and reduced generation of anti-inflammatory metabolites. In parallel, the community often shifts toward mucus-degrading and potentially proinflammatory taxa, including Akkermansia muciniphila (consistently found to be elevated across several studies), Ruminococcus gnavus, Ruminococcus torques, Blautia, Collinsella aerofaciens, and Eggerthella lenta.^14,27,34,35,e13,e14,e16,e19-24,e27,e28^

Although data in progressive MS are limited, available evidence suggests that the same ecological pattern may become more pronounced, with further depletion of SCFA-associated commensals and enrichment of taxa with potentially proinflammatory features. Case–control comparisons in progressive MS have reported enrichment of Enterobacteriaceae-related and other potentially inflammatory taxa, alongside depletion of genera such as Blautia and Agathobaculum.^35^ Other cohorts similarly report depletion of SCFA-associated species, including Ruminococcus bromii and several Faecalibacterium species, together with enrichment of taxa such as Enterococcus faecium.^e13^ Phenotype-focused analyses suggest subtype-specific shifts, including increased Streptococcus with decreased Roseburia in SPMS, and higher Methanobrevibacter and Sporobacter with lower Gemmiger in primary progressive MS (PPMS).^e25,e27^ More recently, enrichment of Tyzzerella nexilis has been reported in SPMS compared with HCs.^e29^ Finally, integrative microbiome–metabolite analyses reinforce the view that progressive trajectories are accompanied by depletion of SCFA-linked taxa (e.g., Eubacterium hallii and Blautia) and enrichment of taxa associated with worsening profiles, including Bacteroides and Alistipes.^36^

Overall, RRMS and predominantly relapsing cohorts show depletion of fiber-degrading and SCFA-associated commensals with enrichment of mucus-degrading/inflammatory taxa, whereas progressive MS datasets suggest that this pattern may persist or intensify, often alongside stronger associations with disability and progression-related phenotypes.

### Conflicting Findings for Specific Taxa

Not all microbiome studies in MS agree, underscoring the complexity of host–microbe interactions and methodological challenges in their interpretation. Akkermansia muciniphila provides a useful conceptual example: Although consistently reported as enriched across multiple MS cohorts, its biological significance remains uncertain.^27,34,35,e11,e19,e20,e27^ One explanation is strain-level heterogeneity because bacteria within the same species may differ in genetic content and functional properties. Consistent with this, Cox et al.^35^ isolated distinct Akkermansia strains from participants with MS and showed differential effects on EAE, supporting strain-specific influences on CNS autoimmunity. In the same study, Akkermansia abundance was inversely associated with disability and MRI burden in progressive MS, suggesting that its enrichment might, in some settings, reflect a compensatory rather than pathogenic response.

A second, nonmutually exclusive explanation is community context dependency. The association of Akkermansia with neuroinflammatory severity may vary depending on the surrounding microbial ecosystem, indicating that the effect of a given organism may not be predictable from its relative abundance alone.^37^ This highlights the importance of considering whole-community networks rather than attributing causality to individual taxa. A third layer of complexity is host dependency. Interactions between host genetics and the gut microbiota can shape disease expression, suggesting that certain bacteria may influence CNS autoimmunity only in genetically susceptible hosts.^38^ Host species specificity further complicates interpretation because microbe–host interactions identified in mouse models may not translate directly to humans. For example, segmented filamentous bacteria colonize different rodent hosts but do not adhere equivalently to the ileal epithelium across species, and because this epithelial adhesion is required for Th17 induction, the resulting immune effect is also host dependent.^39^

Dietary context may further modulate the functional consequences of mucin-degrading bacteria. Under fiber-deprived conditions, the microbiota shifts toward host mucus glycans as a nutrient source, resulting in erosion of the mucus barrier and greater susceptibility to pathogen invasion. Thus, the biological significance of a mucin-degrading taxon such as Akkermansia may depend not only on its abundance but also on whether the dietary environment favors mucus utilization and barrier erosion.^40^ These findings suggest Akkermansia inconsistencies reflect biological complexity rather than poor reproducibility.

Similar challenges extend beyond Akkermansia and are also evident for other taxa repeatedly implicated in MS. Blautia species are frequently enriched in MS and can worsen disease in gnotobiotic models; however, both increases and decreases have been reported for specific members—including B. wexlerae—across cohorts, highlighting intragenus and intraspecies functional heterogeneity (strain-level differences in carbohydrate utilization, capsule polysaccharides, and bile acid metabolism) and the need for species/strain-resolved analyses.^34,35,e11^

Bacteroides provides another example of inconsistent findings, reflecting both functional heterogeneity and limited taxonomic resolution. Some analyses report reduced Bacteroides alongside losses of other fiber-degrading taxa—compatible with diminished polysaccharide breakdown and downstream SCFA production—whereas others report stable or increased abundance, potentially linked to animal protein–/fat-skewed diets and to Bacteroides lineages that preferentially use host glycans/bile acids.^27,33,35,41,e15,e16,e20,e24,e27^ These genus-level averages also mask species/strain divergence. For instance, nontoxigenic Bacteroides fragilis can promote IL-10^+^ Treg responses, whereas enterotoxigenic strains induce Th17 phenotypes.^42,e4^ Because most 16S pipelines cannot resolve strain-level variation, even species-level reports can point in opposite directions.

Anaerotruncus colihominis further illustrates that enrichment does not necessarily imply pathogenicity. Although increased in pwMS relative to HCs in several cohorts,^e20,e23,e28^ it has been shown to ameliorate EAE by inducing Tregs, suggesting that some taxa may reflect compensatory or host-protective responses.^e5^

Several factors likely contribute to inconsistencies across MS microbiome studies. Beyond limited sample sizes, differences in disease stage, clinical subtype, disability level, relapse status, and treatment exposure may influence microbiome composition. Environmental factors such as diet, geography, and lifestyle also shape microbial communities, while control selection (e.g., unrelated vs household controls) introduces additional variability.^34^ Technical differences in sampling, sequencing, and bioinformatic pipelines further complicate interpretation, particularly given the limited taxonomic resolution of many 16S-based approaches. Accordingly, conflicting findings likely reflect both biological context dependence and methodological limitations. Functional approaches—including transplantation studies, gnotobiotic models, metabolomics, and strain-resolved multiomic analyses—will be essential to distinguish causal from compensatory microbial changes and to identify mechanistically relevant features in MS. An overview of current challenges and future directions in the field is provided in Table 2.

**Table 2:** Current Challenges and Likely Technological Advances in MS Microbiome Research Over the Next 5–10 Years

Current challenge	Why it matters	Likely advances in the next 5–10 y	Potential impact on the field
Limited taxonomic resolution	Many studies still rely on 16S rRNA sequencing, which often cannot resolve species-level or strain-level differences. This is particularly problematic when closely related strains exert opposite immunologic effects	Wider use of deep shotgun metagenomics, improved strain-resolved profiling, and more complete reference genome databases	More precise identification of disease-associated microbes and improved distinction between pathogenic, protective, and bystander taxa
Functional ambiguity of taxonomic signals	Changes in microbial abundance do not necessarily reflect biological function, and similar taxonomic profiles may produce very different metabolic or immune outputs	Greater integration of metagenomics with metabolomics, transcriptomics, proteomics, and immune phenotyping	Improved ability to link microbial composition to mechanistic pathways relevant to neuroinflammation and MS progression
Cross-cohort heterogeneity	Differences in geography, diet, lifestyle, treatment exposure, disease stage, and selection of control groups contribute substantially to variability across studies	Better harmonization of study design, metadata collection, and multicenter cohort frameworks, including broader use of paired household controls and standardized protocols	Greater reproducibility across studies and more reliable identification of shared vs population-specific microbiome signatures
Limited longitudinal sampling	Most studies remain cross-sectional, making it difficult to determine whether microbiome changes precede, accompany, or follow disease activity	Expansion of longitudinal sampling across relapse, remission, progression, treatment initiation, and presymptomatic or at-risk phases	Better temporal resolution of microbiome changes and improved ability to distinguish causal from reactive alterations
Incomplete sampling of relevant niches	Stool sampling may not capture mucosal, small intestinal, or other site-specific microbial communities that could be more directly relevant to host immune responses	Increased use of site-specific sampling, spatially resolved microbiome approaches, and paired stool–mucosal analyses	More complete understanding of host–microbe interactions at biologically relevant interfaces
Difficulty distinguishing causality from compensation	Some taxa enriched in pwMS may be disease-promoting, whereas others may reflect compensatory or host-protective responses	Broader use of gnotobiotic models, defined microbial consortia, transplantation studies, organoid systems, and targeted metabolite supplementation approaches	Stronger causal inference and better prioritization of therapeutic targets
Limited integration of host factors	Host genetics, immune state, sex, age, and environmental exposures interact with the microbiome but are often analyzed separately	More integrated host–microbiome study designs combining host genomics, immunophenotyping, environmental exposures, and microbial profiling	Improved understanding of interindividual heterogeneity and identification of microbiome effects that depend on host context
Limited translational validation	Many microbiome findings remain associative and have not yet been translated into biomarkers or therapeutic strategies	Development of microbiome-informed biomarkers, targeted microbial or metabolite-based interventions, and early-phase clinical trials	Greater potential for microbiome-based biomarkers and more rational design of microbiome-based therapies in MS.

Abbreviation: MS = multiple sclerosis.

### Associations of Specific Bacterial Taxa With MS Severity and Progression

While case–control studies consistently show that the gut microbiome of pwMS differs from that of HCs (Section 3.1), efforts to link individual taxa to clinical outcomes—such as disability, relapse activity, MRI burden, or conversion to progressive MS—have yielded more variable and sometimes contradictory results. In RRMS and predominantly relapsing cohorts, several individual taxa have been linked to disability and inflammatory activity. A study reported positive correlations between the Expanded Disability Status Scale (EDSS) and several taxa, including Collinsella aerofaciens, Coprococcus comes, and Sutterella wadsworthensis, together with an inverse association for the SCFA producer Eubacterium siraeum.^33^ Another report identified the “dysbiotic Bacteroides 2 enterotype”—dominated by Bacteroides and depleted in SCFA-producing genera—as a predictor of worsening long-term disability in that cohort, where it outperformed serum neurofilament light as a prognostic markers.^e30^ A longitudinal study reported that baseline signatures characterized by depletion of Akkermansia and SCFA-producing Lachnospiraceae/Oscillospiraceae together with expansion of Alloprevotella, Prevotella-9, and Rhodospirillales were associated with EDSS increase, supporting a possible predictive relationship in that cohort.^e31^ Another longitudinal study reported that higher baseline abundance of butyrate-producing taxa, including Eubacterium hallii, Butyricicoccus, Blautia, and other Lachnospiraceae, as well as Streptococcus thermophilus, was associated with less EDSS worsening, reduced brain atrophy, and better cognition, whereas Alistipes (e.g., A. onderdonkii) and several Bacteroides spp. were associated with greater disability progression and MRI disease burden and, in that cohort, with subsequent conversion from relapsing to progressive disease.^36^ Additional work in RRMS and mixed cohorts further supports associations between relapse activity and shifts in taxa such as Clostridium, Streptococcus, Akkermansia, and key SCFA producers including Eubacterium rectale and Ruminococcus spp.^e25,e32^

In progressive MS, several studies have reported associations between specific microbial features and greater disability or worse clinical trajectories. The iMSMS household-controlled study reported stronger depletion of butyrate producers such as Faecalibacterium prausnitzii and Fusicatenibacter saccharivorans and enrichment of Ruthenibacterium lactatiformans, Hungatella hathewayi, and Eisenbergiella tayi in progressive MS compared with RRMS, with several SCFA-producing Butyrivibrio, Clostridium, and Ruminococcus species inversely associated with disease severity.^34^ Cox et al.^35^ reported that butyrate producers such as Ruminococcus bromii and Roseburia inulinivorans showed inverse associations with disability, and that Akkermansia correlated negatively with EDSS in progressive patients. In an Egyptian cohort, Enterococcus faecium and Clostridium saudiense correlated positively with EDSS, whereas Ruminococcus bromii correlated negatively.^e13^ In a study that combined human microbiome analyses with experimental follow-up, Tyzzerella nexilis lineages enriched in mobile genetic elements were associated with progressive MS, particularly SPMS, and with higher disability in the human cohort, while experimental testing showed that these lineages could exacerbate EAE. These findings provide mechanistic support for a possible contributory role of specific microbial lineages, although causality in humans remains unproven.^e29^

However, these findings should be interpreted cautiously because progressive MS cohorts remain relatively small and may be vulnerable to confounding by treatment exposure, disease duration, and other clinical differences. In addition, geographic and dietary variability may influence these datasets, limiting reproducibility and generalizability of progressive-specific microbial signatures. Together with metagenomic and microbiome–metabolite studies, these findings suggest that functional disruption of SCFA-related and other immunomodulatory pathways may be more closely associated with MS severity and progression than any single taxon, although causal relationships in humans remain to be established.^36^ An important limitation of these observational studies is disease-modifying therapy exposure, which may independently affect microbiome composition and metabolite profiles. In particular, treatment-naïve and treated cohorts should not be assumed to be directly comparable, as ongoing therapies—including first-line agents and B-cell–depleting treatments—may themselves reshape host–microbiome interactions, and variability in the reporting and control of treatment exposure across studies may further complicate cross-cohort comparisons.

Overall, evidence suggests that gut microbiome alterations in pwMS not only distinguish them from HCs but also partially correlate with clinical severity, with a recurring pattern of depleted SCFA-producing commensals and enrichment of potentially proinflammatory taxa in more disabled or progressive patients. However, these associations are not fully consistent across cohorts and do not establish causality in humans, suggesting that severity may reflect broader functional and context-dependent microbial effects rather than a uniform taxonomic signature.

## Microbiota-Derived Metabolites Associated With MS

Focusing on metabolites provides functional insight independent of the specific bacteria present and complements microbiome analyses by identifying biochemical messengers through which the gut microbiota may influence MS. Global and targeted metabolomic studies in MS have revealed perturbations in microbially derived molecules that can modulate host physiology, such as SCFAs, aromatic amino acid metabolites (including tryptophan catabolites), bile acids, and other microbially influenced pathways.^43,44^ Mechanistically, these signals may act through 3 nonmutually exclusive routes: vagus nerve involvement in gut–brain communication, alteration of immune responses at mucosal and systemic levels, and direct CNS penetration with downstream effects on resident glial and neuronal function.^7,45,46^

### SCFAs and Fatty Acid Profiles

SCFAs such as acetate, propionate, and butyrate, produced by gut fermenters, are well-established immunoregulatory mediators. Experimental studies support a role for SCFAs in CNS autoimmunity. In EAE, dietary supplementation with long-chain saturated fatty acids (e.g., lauric acid) worsens disease severity and is associated with reduced intestinal SCFA levels, whereas SCFA supplementation exerts the opposite effect by promoting regulatory immune responses and dampening inflammation. These findings support a mechanistic connection between an unfavorable fatty acid milieu, reduced SCFAs, and enhanced inflammatory potential in experimental models.^47^ At the same time, context may modulate these effects because SCFAs have also been linked to proinflammatory programs under certain conditions, suggesting that concentration, timing, and the surrounding immune milieu likely determine their net impact on MS pathology.^48^

Beyond immune effects, SCFAs may also participate in gut–brain communication through neural pathways. For example, butyrate has been shown to increase jejunal vagal afferent firing, and vagal sensory neurons express the SCFA receptor FFAR3, whose deletion abolishes the effect of propionate on vagal activity, supporting a role for SCFAs in microbiota-dependent neural signaling.^49^ Notably, subdiaphragmatic vagotomy normalizes the abnormal blood metabolite profile observed during cuprizone-induced demyelination, supporting vagal signaling as a conduit for microbiota–brain communication.^7^ Finally, SCFAs can reach the CNS and act on resident cells and barrier function. In EAE, a mixture of acetate, propionate, and butyrate restrains astrocyte activation by enhancing tryptophan–aryl hydrocarbon receptor (AhR) signaling and preserving perivascular AQP4 polarity, thereby stabilizing BBB function and attenuating disease severity.^50^ In toxin-induced demyelination models, butyrate supplementation reduces cuprizone-induced and lysolecithin-induced demyelination and accelerates remyelination by promoting oligodendrocyte precursor cell maturation.^51^

In pwMS, SCFAs have frequently been reported at altered levels in serum and/or feces, although findings are not uniform across disease stages and cohorts. Multiple studies report reduced propionate and butyrate in serum and/or feces of pwMS, together with a depletion of butyrate-producing taxa, whereas acetate shows a more heterogeneous pattern, with some cohorts showing reduced levels and others increased levels that correlate with higher disability and proinflammatory signatures.^48,52,53,e33,e34^ Notably, fecal SCFA depletion may not be evident at disease onset because treatment-naïve patients sampled at first acute RRMS relapse had fecal SCFA levels comparable with HCs, with reductions mainly observed in long-standing RRMS receiving disease-modifying therapies.^54^ Consistent with a possible association with chronicity and progression, acetate, propionate, and butyrate have been reported to be decreased in active SPMS with long disease duration, and dysregulated lipid profiles have also been associated with the development of progressive MS.^36,48^

Moreover, colon-derived SCFAs can enter the circulation and exert broad immunomodulatory effects, including enhancing Treg-cell generation, migration, and function.^47,48,52^ In pwMS, oral propionate supplementation has been reported to increase circulating Treg frequencies, reduce proinflammatory Th17 responses, and be associated with fewer relapses and favorable MRI and neurodegeneration-related markers.^47^ Propionate has also been detected in the CSF of pwMS, and supplementation has been associated with changes in gray matter volume and neurodegeneration-related markers.^52^

Together, these findings position SCFAs as plausible pleiotropic mediators linking gut microbial activity to immune regulation, gut–brain communication, and barrier stability in MS. Experimental models show that SCFAs influence disease through multiple mechanisms, including immune modulation, neural signaling, and effects on CNS-resident cells. Human studies indicate that SCFA homeostasis is altered in at least a subset of pwMS and that restoration of specific SCFAs, particularly propionate, may have clinical and immunologic benefits. Thus, SCFAs represent one of the clearest examples of a microbiota-derived metabolite class for which associative human observations and mechanistic animal data converge, although causality in pwMS remains to be fully established.

### Tryptophan and Aromatic Amino Acid Metabolism

Tryptophan is extensively metabolized by gut bacteria into metabolites with context-dependent effects on CNS inflammation. Experimental studies support functional consequences of dysregulated microbial tryptophan metabolism in CNS autoimmunity. Indole metabolites such as indole lactate attenuate neuroinflammation and promote remyelination in experimental models.^55^ In parallel, microbial transformation of dietary tryptophan generates diverse AhR ligands and related metabolites that shape immune responses and can directly modulate CNS-resident cells. Several indole derivatives signal through AhR in microglia and astrocytes, dampening neuroinflammation.^9,56^ In EAE, both dietary tryptophan availability and the composition of tryptophan-metabolizing bacterial communities have been shown to tune the encephalitogenicity of autoreactive T cells, shifting the balance between pathogenic Th17 responses and regulatory programs.^e35,e36^ By contrast, microbiota-derived kynurenic acid can recruit GPR35^+^ Ly6C^+^ macrophages that support Th17 responses in the small intestine, thereby enhancing CNS autoimmunity.^e37^ Together, these studies indicate that specific tryptophan-derived metabolites can exert either protective or pathogenic effects depending on the metabolic context and the downstream host pathways engaged.

Tryptophan metabolism is markedly altered in pwMS. Tryptophan is metabolized through 3 main routes—the kynurenine, serotonin, and indole pathways—and integrated host–microbial profiling shows that microbial tryptophan catabolites are differentially regulated in pwMS compared with HCs.^44^ Targeted serum metabolomics indicates that pwMS exhibit reduced circulating tryptophan and 5-hydroxytryptophan (5-HTP, the immediate precursor of serotonin), together with increased downstream kynurenine-derived, serotonin-derived, and indole-derived metabolites, consistent with enhanced tryptophan catabolism toward neuroactive and immunoactive products.^e38^ An integrated microbiome–metabolome study also reported reduced levels of the microbiota-derived tryptophan metabolites indolelactate and indolepropionate in pwMS, suggesting loss of potentially neuroprotective and anti-inflammatory microbial indoles.^57^ At a broader scale, a large multiomic study showed that pwMS have reduced levels of lactate-derived aromatic metabolites that can act as immunoregulatory agonists of the AhR and increased levels of gut-derived aromatic metabolites (e.g., indole acetic acid [IAA] and p-cresol derivatives) associated with higher disability and reduced AhR pathway activity, indicating a shift toward a more proinflammatory aromatic amino acid profile in MS.^44^ A recent longitudinal multiomic study further found that subjects who later transitioned to progressive MS displayed a characteristic disturbance in microbial aromatic amino acid metabolism, with increased serum p-cresol sulfate and decreased fecal phenyl acetate and nicotinate, consistent with loss of potentially protective aromatic amino-acid–related metabolites and accumulation of harmful aromatic products.^36^

Beyond quantitative shifts in tryptophan availability and beneficial indoles, several studies have focused on potentially neurotoxic aromatic metabolites. A study integrated plasma and CSF metabolomics in RRMS, reporting that certain metabolites arising from tryptophan and phenylalanine catabolism (p-cresol sulfate and indoxyl derivatives) were shown to impair neuronal function in vitro and were associated with neurofilament light chain and cortical atrophy in pwMS, further implicating dysregulated aromatic amino acid metabolism in MS neurodegeneration.^46^ Notably, in the same experimental-human translational framework, indole lactic acid (ILA) also restored a dysregulated ILA:IAA balance, and a lower ILA:IAA ratio was associated with worse clinical outcomes in pwMS.^55^

Together, these findings support a role for microbial tryptophan metabolism in MS pathophysiology through effects on immune regulation, CNS-resident cells, and remyelination. Human studies associate altered aromatic metabolite profiles with disability, neurodegeneration-related markers, and progression, whereas experimental models demonstrate functional effects of specific tryptophan-derived metabolites on CNS autoimmunity.

### Bile Acids and Related Pathways

Bile acids are a major class of microbiota-influenced metabolites altered in MS. Experimental studies show that bile acids modulate CNS autoimmunity through immunologic and neuroactive mechanisms. Secondary bile acids have been shown to promote Treg-cell differentiation at the expense of Th17 programs and restrain CNS autoimmunity.^58^ Bile acids can also cross the BBB and modulate glial activation states.^45,59^ Consistent with these mechanisms, experimental work demonstrates that tauroursodeoxycholic acid (TUDCA) ameliorates neuroinflammation and modulates glial activation in EAE.^45,58^

In pwMS, metabolomic studies reveal altered bile acid profiles, with changes in primary and secondary bile acid species indicative of disrupted host–microbiome metabolism.^45^ Bile acid alterations have been linked to clinical outcomes in distinct cohorts of pwMS: Higher baseline primary bile acid levels were reported to predict slower brain and retinal atrophy in 1 study, whereas marked depletion of secondary bile acids in stool was associated with disease progression in another.^36,59^ In pwMS with progressive disease, TUDCA supplementation has been reported to increase circulating bile acids and to induce measurable immunologic and gut microbiome changes.^59^Together, these findings support bile acid dysregulation as a functional axis linking gut microbial metabolism to immune balance and CNS-resident inflammatory programs in MS.

### Additional Gut-Derived Metabolites Relevant to MS

Beyond SCFAs, tryptophan derivatives, and bile acids, additional gut-derived metabolites may also influence MS-related neuroinflammation, although the evidence remains limited and predominantly experimental. In a gnotobiotic EAE model, microbiota-derived γ-aminobutyric acid was enriched in Akkermansia-containing communities and correlated with greater disease severity.^37^ In a separate experimental study, a ketogenic diet increased β-hydroxybutyrate and promoted indole-3-lactate–producing Lactobacillus strains, suggesting that diet-shaped host metabolism can also modulate autoimmune neuroinflammation.^60^ Together, these findings point to a broader, still-emerging metabolomic layer beyond the major SCFA, tryptophan, and bile acid axes.

Across these metabolite classes, several common mechanistic themes emerge. SCFAs, tryptophan-derived metabolites, and bile acids seem to converge on a limited number of shared host pathways despite their biochemical diversity. They can all influence immune and neuroglial responses through overlapping signaling nodes, including G protein-coupled receptor signaling, AhR-dependent pathways, and vagal or other gut–brain communication routes. Across studies, they repeatedly affect astrocyte and microglial phenotypes and modulate barrier integrity at both the intestinal and blood–brain interfaces. This suggests that microbiota-derived metabolites act not as isolated factors, but as an interconnected network linking gut ecology to immune regulation and neuroinflammation in MS.

## Conclusion

Together, current microbiome and metabolomic evidence firmly positions the gut as a relevant compartment in MS, not through a single “MS microbe” but through coordinated alterations in microbial communities and their metabolic output. Recurrent patterns of microbial dysbiosis and disturbed SCFA, tryptophan, and bile acid pathways support plausible routes linking gut dysbiosis to immune regulation, barrier integrity, and CNS-resident cell function. However, most human studies remain observational, limiting causal inference. Cross-sectional studies can identify associations with disease status, disability, or progression, and longitudinal cohorts may reveal predictive relationships in specific settings, but such findings do not by themselves establish causality. Although mechanistic support from experimental systems strengthens biological plausibility, such findings cannot be assumed to fully recapitulate causal relationships in humans. Future work will require longitudinal, mechanistically integrated studies across gut, blood, and CNS to distinguish disease-driving processes from secondary or compensatory changes. If these challenges are met, the gut microbiota–metabolite axis may provide biomarkers and therapeutic targets for MS.

## Glossary

AhR: aryl hydrocarbon receptor
BBB: blood–brain barrier
EAE: experimental autoimmune encephalomyelitis
EDSS: Expanded Disability Status Scale
FMT: fecal microbiota transplant
GF: Germ-free
HC: healthy control
MS: multiple sclerosis
pwMS: people with MS
RRMS: relapsing–remitting MS
SCFA: short-chain fatty acid
SPMS: secondary progressive MS
TUDCA: tauroursodeoxycholic acid
